# Design and Validation of the PercOV-S Questionnaire for Measuring Perceived Obstetric Violence in Nursing, Midwifery and Medical Students

**DOI:** 10.3390/ijerph17218022

**Published:** 2020-10-30

**Authors:** Desirée Mena-Tudela, Agueda Cervera-Gasch, María José Alemany-Anchel, Laura Andreu-Pejó, Víctor Manuel González-Chordá

**Affiliations:** 1 Department of Nursing, Faculty of Health Sciences, Universitat Jaume I, Avda. Sos I Baynat s/n, 12071 Castellón, Spain; cerveraa@uji.es (A.C.-G.); pejo@uji.es (L.A.-P.); vchorda@uji.es (V.M.G.-C.); 2 Midwives’ Teaching Unit of the Comunitat Valenciana, 46017 Valencia, Spain; alemany_marjos@gva.es

**Keywords:** obstetric violence, gender violence, childbirth, birth, health sciences students

## Abstract

Background: Obstetric violence could be defined as the dehumanized treatment or abuse of health professionals towards the body or reproductive process of women. Some practices associated with obstetric violence have been routinely standardized and do not include the woman in decision making. This type of violence has consequences for the health of both the mother and the baby and that of the professionals who practice or observed it. Methods: A questionnaire consisting of 33 items that measured perception through a Likert scale was developed. Some sociodemographic variables were collected. The instrument was applied to a sample of nursing, medicine and midwifery students to determine its psychometric properties. Results: The final sample consisted of 153 students. The Kaiser-Meyer-Olkin (*p* = 0.918) and Barlett tests (*p* ≤ 0.001) allowed for factor analysis, which explained 54.47% of the variance in two factors called protocolized-visible obstetric violence and non-protocolized-invisible obstetric violence. Conclusions: The PercOV-S (Perception of Obstetric Violence in Students) instrument was validated. The distribution and content of the two factors are closely related to obstetric violence against women. The existence of statistically significant relationships between the sociodemographic variables collected and the global measurements, domains and items of the PercOV-S scale highlight the normalization of obstetric violence as a central factor for future studies.

## 1. Introduction

Some definitions of Obstetric Violence (OV) exist, although the United Nations Population Fund (UNFPA) has recognized that there is a lack of consensus on how to define and measure violence against women during delivery care in health centres [1]. However, some South American countries have legislated around OV and provided a definition. For example, Venezuela defines this term as “the appropriation of the body and reproductive processes of women by health personnel, which is expressed as dehumanized treatment, an abuse of medication and the conversion of natural processes into pathological ones, bringing with it loss of autonomy and the ability to decide freely about their bodies and sexuality, negatively impacting the quality of life of women” [2].

The World Health Organization (WHO) describes some practices that make OV visible: disrespectful and offensive treatment during childbirth, physical abuse, profound humiliation and verbal abuse, the performance of medical procedures without consent or with coercion (including sterilization), lack of confidentiality, failure to obtain full informed consent, refusal to administer analgesics, flagrant violations of privacy, rejection of admission to a health centre, neglect of women during childbirth, and retaining women and newborns at a health centre due to their inability to pay [3], among issues. In 2015, the International Federation of Gynaecology and Obstetrics (FIGO) proposed the Mother and Baby Friendly Birth Facility (MBFBF), which identified 7 categories of disrespect and abuse in childbirth [4]. In these categories, examples of obvious abuse, such as hitting the woman; forcing her legs apart; placing pressure on the fundus; not providing privacy (spatial, visual or auditory); humiliation; screaming at or demeaning the woman; and discrimination based on ethnic origin, age, marital status, language, economic status or educational level, among other violations, were exposed [4].

Studies identify that approximately 30% of childbirth experiences can be considered traumatic and/or as having OV components [5,6]. Moreover, OV can be found around the world in all continents: America [7,8,9], Africa [10,11,12], Asia [13], Europe [6,14,15] and Australia [16,17]. These negative experiences and the presence or practice of OV have consequences for the woman and her newborn, for society and for the health professionals who observe or practice it. Among the main consequences for women, the impact on physical, sexual and psychological health can be highlighted [5,18]; specific examples are the loss of the uterus after an unnecessary c-section or permanent pain during intercourse with penetration after an episiotomy [19]. For health personnel, the most prominent consequence is secondary traumatic stress and compassionate fatigue [20] that may even lead professionals to abandon professional practice [21].

Many of the health interventions related to OV are normalized; therefore, health personnel may not even be aware that they are engaging in violent practices against a woman [22]. Scientific literature reveals that the factors that perpetuate OV include a lack of proper training for health professionals [1,23]. With respect to health science students, although the literature highlights the importance of providing high-quality woman-centred care, many forms of lack of respect and abuse during childbirth continue to be reported [24]. With all this, the negative obstetric practices and the existing literature make us think about an important public health problem: obstetric violence. Incorporating techniques to prevent obstetric violence seems necessary. Making health science students aware of this problem can be part of the solution to this problem. Among this literature, no validated instruments have been identified to measure the perception of OV by health science students. Thus, with this study, we intend to construct and validate an instrument (the PercOV-S, Perception of Obstetric Violence in Students) that can evaluate the health sciences students’ perceptions regarding certain practices related to obstetric violence.

## 2. Materials and Methods

### 2.1. Design

An instrumental design was used in this study [25] to develop and validate an instrument that would allow the study of health sciences students’ perceptions of obstetric violence (PercOV-S). The study was conducted between November 2018 and October 2019. The study was approved by the Management of the Nursing Research Group (241) of the Universitat Jaume I, and the Deontological Committee of the Universitat de Jaume I (CD/26/2020) approved this study. The project was designed in accordance with the Organic Law 03/2018 of December 5, Protection of Personal Data and Guarantee of Digital Rights. In addition, the principles of the Declaration of Helsinki were respected.

### 2.2. Setting and Participants

The study was conducted with nursing, midwifery and medical students. Nursing and medical students belong to the Universitat Jaume I (Spain). Midwifery students are from the Valencian School of Health Studies (EVES). The established sample size was 5 subjects per item, according to the recommendations of the literature [26]. All students enrolled in the nursing or medical program during academic year 2018/2019 at the Universitat Jaume I (Spain) and who attended an educational seminar on OV were eligible. All midwifery students present in the classroom on the day of data collection were included. Finally, the questionnaire was administered to a sample of 169 nursing, medical and midwifery students to determine the psychometric properties of the instrument.

### 2.3. Instruments Validation and Collection

The development and validation of the PercOV-S instrument were carried out according to the recommendations of the literature [27]. First, a literature review was conducted to confirm that no other instruments had been developed specifically for the same aim in the last 5 years. The PubMed, Cochrane databases, and Cumulative Index to Nursing and Allied Health Literature were consulted, 5-year-old articles in Spanish and English were included. The Spanish Clinical Practice of Normal Birth Care Guide was consulted [28,29]. This review was used to determine which interventions could be related to obstetric violence. The first version, obtained by a group of 3 members of the research group, comprised 33 items measured with the Likert scale (between November and December 2018).

In the next step, content validity was studied. For content validation, a heterogeneous group of experts in health and gender composed of a graduate in medicine, a nurse, a lawyer and a mother with previous experience with OV was formed. The experts received the scale 72 h before the meeting by e-mail. A single online meeting was necessary to reach agreement, and no items were changed (between December 2018 and January 2019).

Finally, the questionnaire was administered to nursing and medical students in March 2019. Midwifery students received the questionnaire from March to June 2019. To collect the data, the paper-based questionnaire was administered to nursing and medical students before an educational seminar on obstetric violence. For midwifery students, the questionnaire was sent online before the training.

### 2.4. Measurement

The final questionnaire consisted of 33 items that measured the students’ perceptions regarding OV using a 5-level ascending Likert scale (1: no OV; 5: a considerable amount of OV). All items were positively written. Five points can be obtained for the maximum perception of obstetric violence for each item. Higher scores indicate an increased perception of obstetric violence. In addition, the following sociodemographic variables were collected and analysed: age, gender (male/female), area of study (nursing, medicine and midwifery), health experience in gynaecology and obstetrics services (yes/no), witnessing a birth (yes/no), and personal experience with pregnancies and births (yes/no).

### 2.5. Data Analysis

First, the internal consistency of the questionnaire and its domains were determined with Cronbach’s alpha. To study construct validity, the viability of the exploratory factor analysis was confirmed with the Kaiser-Meyer-Olkin test (KMO = 0.918) and Bartlett’s test of sphericity (*p* ≤ 0.001). Factor extraction was performed using principal components with varimax rotation. Second, a descriptive analysis of the sample was performed according to the nature of the variables (mean, standard deviation, maximum and minimum, and 95% confidence intervals were determined for quantitative variables, and frequency and percentage were determined for qualitative variables). Finally, the questionnaire results were analysed descriptively, and statistically significant relationships between sociodemographic variables and perceptions of OV were determined with the Mann–Whitney U test and the Kruskal–Wallis test. Calculations were performed using SPSS for Windows, and the level of statistical significance was established at *p* ≤ 0.05.

## 3. Results

### 3.1. Sociodemographic Characteristics

A total of 169 questionnaires were collected, of which 9.46% (n = 16) were eliminated due to poor completion. The sample consisted of 153 questionnaires. The distribution of the sample according to area of study was as follows: nursing students, 60.8% (n = 93); medical students, 9.2% (n = 14); and midwifery students, 30.1% (n = 46). The mean age of the sample was 23.34 years (±5.426; minimum = 19, maximum = 61). A total of 89.5% (n = 137) of the sample was female. Forty percent (n = 62) of the sample had worked or completed internships at gynaecology and obstetrics services; of these, 38.7% (n = 24) had done so less than a year ago, 51.6% (n = 32) had done so between 1 and 4 years ago, and 9.7% (n = 6) had done so more than 4 years ago. A total of 39.2% (n = 60) of the students had witnessed a childbirth; of these, 90.3% (n = 56) had done so between less than 1 year and 4 years ago. A total of 5.9% (n = 9) were pregnant, and 3.9% (n = 6) had given birth (Table 1).

### 3.2. Psychometric Properties

There were no comprehension problems with the items. The viability of the factor analysis was confirmed by the KMO test (*p* = 0.918) and Bartlett’s test for sphericity (*X^2^* = 3767.745; *p* ≤ 0.001). The factor analysis explained 54.47% of the variance in the two factors. The first factor or domain explained 43.11% of the variance and comprised 8 items. The second factor or domain explained 11.36% of the variance and comprised 25 items. All items showed factor loads greater than 0.4 except “Allowing skin-to-skin contact after the paediatric examination”, although the research team decided to keep it to obtain relevant information on a specific procedure. The overall reliability of the questionnaire (α = 0.936) and its domains was excellent (domain 1, α = 0.802; domain 2, α = 0.952), and it was decided not to eliminate any item by increasing the α value. Table 2 shows the results of the exploratory factor analysis and reliability tests.

### 3.3. Student Perception Scale of Obstetric Violence

The overall mean score of the questionnaire was 4.14 (SD ± 0.61; 95% CI = 4.03–4.23), indicating a high perception of obstetric violence. The domain 1 score obtained a mean score of 3.17 (SD ± 0.86; 95% CI = 3.03–3.32); for domain 2, the mean score was 4.25 (SD ± 0.61; 95% CI = 4.15–4.35).

Regarding inferential analysis, the overall PercOV-S score showed some statistically significant relationships to the sociodemographic variables sex (male: m = 3.68, ± 0.95; female: m = 4.18, ± 0.52, *p* = 0.029); area of study (nursing: m = 4.07, ± 0.56; medicine: m = 4.13, ± 0.48; and midwifery: m = 4.25, ± 0.66, *p* = 0.011) and completion of internships in an obstetrics and gynaecology department (yes: m = 4.22, ± 0.58; no: m = 4.06, ± 0.60, *p* = 0.019). Domain 1 showed a statistically significant difference by gender (male: m = 3.71, ± 1.08; female: m = 4.31, ± 0.50, *p* = 0.004), while domain 2 showed a significant effect of area of study (nursing: m = 3.00, ± 0.88; medicine: m = 3.00, ± 1.05; and midwifery: m = 3.53, ± 0.68, *p* = 0.004) and completion of an internship in the gynaecology and obstetrics department (yes: m = 3.43, ± 0.82; no: m = 2.98, ± 0.85, *p* = 0.003) (Table 3). Table 4 shows the results of the PercOV-S items according to the area of study.

## 4. Discussion

In the present study, the psychometric properties of the PercOV-S instrument, which assesses health sciences students’ perceptions in relation to OV, were explored. The internal reliability of the instrument was excellent, and the factor analysis explained 54.47% of the variance in 2 domains that could be defined as (domain 1) non-protocolized-invisible OV and (domain 2) protocolized-visible OV. Observing the factorial load of each of the items, its distribution in each of the domains and the number of items associated with each of the domains, it is easy to see that this type of violence against women may represent the tip of an iceberg model. As other reports of violence against women have shown, a very substantial part of this type of violence remains hidden [30] and it is difficult to identify for various reasons. OV, therefore, as a type of violence against women, cannot be represented in any other way.

Regarding the protocolized-visible OV (domain 2), it can be observed that all items associated with this domain are interventions that are usually protocolized in health centres. This domain has been called visible given that these practices have been the norm for a long time, and even today, they remain in force in many health centres despite not being recommended by scientific evidence and woman-focused care [29]. The WHO already recognized that “*Studies have shown that a substantial proportion of healthy pregnant women undergo at least one clinical intervention during labour and birth, such as labour induction, oxytocin augmentation, caesarean section, operative vaginal birth or episiotomy. In addition, women in labour continue to be subjected to ineffective and potentially harmful routine interventions, such as perineal shaving, enemas, amniotomy, intravenous fluids, antispasmodics and antibiotics for uncomplicated vaginal births. This interventionist approach is not adequately sensitive to the woman’s (and her family’s) personal needs, values and preferences and can weaken her own capability during childbirth and negatively impact her childbirth experience*” [31]. Regarding certain practices as a standard or protocol contributes to their normalization without taking into account aspects as basic as the woman’s own opinion or decisions [22,32]. In addition, this normalization can be seen when comparing the results obtained in this study for domain 1 and domain 2 of the instrument; this comparison showed a notably lower mean for domain 2 (visible) and notably lower scores for the items related to this domain. Consequently, it is not surprising that these interventions are performed on women who come to a health centre to give birth. In other words, normalization of these interventions seems to be present.

On the other hand, according to some authors, the lack of respect for women in delivery rooms has become a dynamic reflecting a lack of ethics and professionalism, which has led to ever-increasing investigations into the status of certain obstetric practices [33,34]. Regarding this point, a large proportion of non-protocolized or invisible OV was identified in this study. This broad factor encompasses practices such as lack of respect, violent language or the use of prohibited practices, such as the performance of the Kristeller manoeuvre; however, although it is known that such practices occur in delivery rooms, they are difficult to identify due to factors such as the lack of recording of the clinical histories of births [35]. Regarding this factor, it is worth noting the low factorial load of the item related to allowing “skin-on-skin contact after the paediatric examination”, which the authors decided to maintain nonetheless since it is the only item that refers to this practice. After long reflection, the authors consider that this item may need a separate domain due to the importance and consequences of this practice on the physical and psychological health of both the mother and the baby and on the link between the two [36].

Regarding the sample, the high percentage of female participants (89.5%) is notable; it may have been motivated by the topic being addressed or due to the fact that female representation in the health sciences is increasing [37,38]. The obtained results show that women perceive OV more than men in a wide range of interventions, possibly because they already have identified some gynaecological or obstetric practices as unpleasant due to their own experience in this area. Therefore, they may be more sensitized [12].

When the results were compared in terms of the participants’ area of study, it was observed that the midwifery students perceived more OV in many of the items of the instrument; the same observation was true for the total score and the non-protocolized-invisible. It is noteworthy that midwifery students scored highest on items such as “not offering measures to treat pain”, but it can be understood that, from a salutogenesis approach, the process of pregnancy and childbirth is studied as a physiological process and health process in women’s sexual and reproductive life [39]; therefore, it may be that offering some measures to treat pain disrupts the physiology of these processes [40]. Other items that were statistically significantly higher among the midwifery students and where midwives perceived more OV were related to performing the Kristeller manoeuvre, performing an episiotomy without anaesthesia, and not allowing the woman to be accompanied if the delivery is instrumented and if a caesarean section will be performed. It is known that, in Spain, the Kristeller manoeuvre is performed in 25% of vaginal births and that, despite being recognized by professionals as a practice that is not free of risks (which are unilaterally assumed), it is practised in secret and health care providers describe how they learn and practice this manoeuvre [35]; therefore, it is evident that it is still being learned and put into practice in Spanish delivery rooms. For episiotomy without anaesthesia, midwives may feel that an episiotomy should only be performed if there is clinical need and that anaesthesia should only be administered in cases of emergency due to acute foetal compromise [29] based on the salutogenesis approach highlighted above. Finally, with regard to accompaniment in cases of instrumentation or caesarean section, it is known that nurses and midwives should be the guardians and guarantors of women’s rights [12], but when these interventions are carried out, gynaecology and anaesthesia professionals become involved, shifting the role of the midwife and nurse to the background [24] and perhaps impacting the continuity of care and the woman’s satisfaction with that care [41].

With all these findings, it seems that health science students have a role that may be important in the detection and prevention of obstetric violence. Being able to detect this type of violence, learning how to treat women from a comprehensive perspective and integrating this knowledge from the beginning of their learning can be essential parts of preventing obstetric violence.

Regarding PercOV-S results according to whether the participant had undergone internships in obstetrics-gynaecology departments and had attended a birth, it was striking that, for the overall score and the non-protocol-invisible domain, students who had participated in internships and/or who had witnessed a birth were better able to perceive OV than students who had not been on rotation for these services. The fact that students are able to identify certain practices as OV does not mean that they are on track to addressing this type of violence. Although identification is a beginning, one must also be aware that students also learn how to justify this learned violence, lack of respect, and abuse in delivery and maternity wards [24]. This presents a broad field for future lines of research in this area, with a fundamental role being played by the development of strategies and learning plans related to any violence against women, for example, domestic violence during pregnancy [42].

Finally, it should be noted that, although there were statistically significant differences according to the participants’ personal experience with pregnancies and births for a considerable number of items on the PercOV-S scale, it was the portion of the sample that had no experience that scored highest in the global score, the visible domain, the invisible domain and some specific items. This result is very striking since it can be associated with the normalization of OV during the sexual and reproductive life of women, which leads to certain practices not being perceived as violent or abusive [43]. However, it is also true that these results should be interpreted with caution since they may be due to the limited representativeness of the sample in terms of the evaluated sociodemographic aspects. Other limitations of the study may be associated with not having conducted a panel of experts with a robust methodology such as that proposed by Polit et al. [44] for content validity of the PercOV-S instrument, even so the results seem consistent. Although registering 5 subjects per item is appropriate, the final sample of the study was too adjusted; this sample should be larger in future studies. Finally, another limitation of the study may be related to the impossibility of performing a temporal stability test, although this test is expected to be carried out in future studies as well as a confirmatory study of the factorial analysis; therefore, the results of this study should be taken with caution.

## 5. Conclusions

The psychometric properties of the PercOV-S instrument, which assesses health sciences students’ perceptions in relation to OV, were explored. According to the results of this study, the instrument comprised 33 items rated on a Likert scale. The constructed instrument has a high degree of reliability and internal validity. Construct analysis yielded adequate results, and factor analysis indicated that 54.47% of the variance of the instrument was explained through 2 domains: (1) protocolized-visible OV and (2) non-protocolized-invisible OV. The distribution and content of these two factors are considered closely related to this type of violence against women, which can be compared to an iceberg in which only a small proportion is visible and a large mass is hidden. Specific training for health professionals in relation to obstetric violence is necessary, as it can be a key element in the prevention of this type of violence. On the other hand, the existence of statistically significant relationships between the sociodemographic variables collected and the global measurements according to the domains of the PercOV-S scale, was observed, highlighting the need to focus on normalization of OV as a central factor in future studies.

## Figures and Tables

**Table 1:** Sociodemographic characteristics (N = 153).

	Nursing Students		Medical Students		Midwifery Students		Overall Score	
	(n = 93)		(n = 14)		(n = 46)		(N = 153)	
	n	%	n	%	n	%	n	%
Gender								
Male	10	10.8	1	7.1	5	10.9	16	10.5
Female	86	89.2	13	92.9	41	89.1	137	89.5
Obstetrics-gynaecology practical experience								
Yes	25	26.9	5	35.7	32	69.6	62	40.5
No	68	73.1	9	64.3	14	30.4	91	59.5
Witnessed a birth								
Yes	20	21.5	2	14.3	38	82.6	60	39.2
No	73	78.5	12	85.7	8	17.4	93	60.8
Personal experience with pregnancy								
Yes	5	5.4	-	-	4	8.7	9	5.9
No	88	94.6	14	100	42	91.3	144	94.1
Personal experience with birth								
Yes	3	3.2	-	-	3	6.5	6	3.9
No	90	96.8	14	100	43	93.5	147	96.1

**Table 2:** Matrix of components and results of the factor analysis of the PercOV-S (Perception of Obstetric Violence in Students) instrument (N = 153).

Domains and Items	Factors			Communalities
	1	2	*α*	
**Domain 1**			0.802	
1. Inserting an intravenous channel	−0.084	0.507	0.939	0.558
2. Directing the woman’s position	−0.023	0.743	0.940	0.569
3. Accelerate the birthing process artificially	0.391	0.608	0.935	0.680
4. Administering routine enemas	0.450	0.597	0.934	0.601
6. Performing routine genital shaving	0.526	0.593	0.933	0.662
15. Enforcing the lithotomy position (i.e., position in which the patient is supported on her back with her legs resting on the stirrups of a surgical table or chair)	0.374	0.601	0.935	0.684
16. Allowing accompaniment during the second stage	0.146	0.452	0.939	0.529
28. Immediately cutting the cord	−0.412	0.491	0.934	0.652
**Domain 2**			0.952	
5. Performing routine amniorrhexis (i.e., artificial rupture of membranes)	0.612	0.313	0.933	0.677
7. Immobilizing the woman	0.731	0.148	0.932	0.615
8. Performing a pelvic exam without consent	0.901	−0.255	0.932	0.896
9. Not offering measures for pain	0.768	−0.197	0.933	0.681
10. Encouraging the use of an epidural	0.639	0.388	0.932	0.606
11. Not preserving privacy	0.894	−0.150	0.932	0.833
12. Convincing the woman to undergo a c-section to end labour quickly and without pain	0.748	−0.015	0.933	0.634
13. Not considering the woman’s decision	0.865	−0.226	0.933	0.808
14. Taking pictures without permission	0.901	−0.267	0.932	0.897
17. Performing routine episiotomy (i.e., incision in the perineum of a woman in labour)	0.620	−0.078	0.934	0.567
18. Saying “You do not know how to push”	0.808	−0.234	0.933	0.757
19. Performing the Kristeller manoeuvre (i.e., fundal pressure in second stage labour)	0.543	−0.013	0.934	0.643
20. Performing an episiotomy without anaesthesia	0.636	−0.148	0.934	0.629
21. Prohibiting eating and drinking	0.444	0.351	0.934	0.545
22. Not providing covering/heating during delivery	0.767	−0.105	0.933	0.608
23. Saying “Stop complaining; it is not that bad”	0.854	−0.320	0.933	0.839
24. Not letting the woman shout	0.740	−0.015	0.932	0.563
25. Performing a caesarean section due to slow dilation	0.627	0.138	0.933	0.491
26. Performing an emergency caesarean section without consent	0.685	0.072	0.933	0.654
27. Not allowing accompaniment in cases of instrumentation or caesarean section	0.715	−0.102	0.933	0.622
29. Suturing a tear without anaesthesia	0.757	−0.087	0.933	0.664
30. Separating the mother and newborn	0.866	−0.280	0.933	0.860
31. Allowing skin-to-skin contact after the paediatric examination	0.293	0.110	0.937	0.589
32. Taking the baby to the nursery	0.570	0.192	0.934	0.507
33. Giving formula without the mother’s consent	0.846	−0.216	0.932	0.810

**Table 3:** Descriptive and comparative results of the sociodemographic variables and PercOV-S.

	Global			Domain 1			Domain 2		
	*M*	*SD*	*p* ^1^	*M*	*SD*	*p* ^1^	*M*	*SD*	*p* ^1^
Sex			0.029			0.661			0.004
Male	3.68	0.95		3.08	0.98		3.71	1.08	
Female	4.18	0.52		3.18	0.85		4.31	0.50	
Area of study									
Nursing	4.07	0.56	0.011 ^2^	3.00	0.88	0.004 ^2^	4.23	0.56	0.182 ^2^
Medicine	4.13	0.48		3.00	1.05		4.32	0.33	
Midwifery	4.25	0.66		3.53	0.68		4.29	0.72	
Obstetrics-gynaecology practical experience									
Yes	4.22	0.58	0.019	3.43	0.82	0.003	4.29	0.56	0.318
No	4.06	0.60		2.98	0.85		4.23	0.63	
Witnessed a birth									
Yes	4.17	0.67	0.099	3.32	0.83	0.116	4.25	0.69	0.191
No	4.10	0.54		3.07	0.87		4.25	0.53	
Personal experience with pregnancy									
Yes	3.66	1.20	0.471	3.01	1.26	0.727	3.71	1.25	0.429
No	4.16	0.52		3.18	0.83		4.29	0.52	
Personal experience with birth									
Yes	3.33	1.30	0.143	2.75	1.08	0.301	3.38	1.43	0.189
No	4.16	0.53		3.19	0.85		4.29	0.52	

^1^ Mann–Whitney U test. ^2^ Kruskal–Wallis test.

**Table 4:** Descriptive and comparative results of the area of study and PercOV-S items.

	Area of Study						
	Nursing		Medicine		Midwifery		*p* ^1^
	*M*	*SD*	*M*	*SD*	*M*	*SD*	
Inserting an intravenous channel	2.12	1.16	2.38	1.32	2.11	0.99	0.747
Directing the woman’s position	2.09	1.33	2.36	1.55	2.74	1.54	0.051
Accelerating the birthing process artificially	3.34	1.30	3.36	1.39	4.02	1.06	0.010
Administering routine enemas	3.18	1.35	2.71	1.43	4.02	1.16	≤0.001
Performing routine amniorrhexis	4.12	0.90	3.86	1.16	4.20	1.02	0.471
Performing routine genital shaving	3.42	1.43	2.77	1.36	4.28	1.08	≤0.001
Immobilizing the woman	4.51	0.84	4.36	0.92	4.67	0.89	0.085
Performing a pelvic exam without consent	4.78	0.73	4.93	0.26	4.83	0.82	0.462
Not offering measures for pain	4.43	0.85	4.86	0.36	4.74	0.88	0.001
Encouraging the use of an epidural	3.93	0.99	4.00	1.03	4.41	0.88	0.009
Not preserving privacy	4.76	0.73	5.00	0.00	4.76	0.84	0.313
Convincing the woman to undergo a caesarean section to end labour quickly and without pain	4.45	0.95	4.64	0.49	4.74	0.85	0.031
Not considering the woman’s decision	4.75	0.65	4.93	0.26	4.78	0.84	0.310
Taking pictures without permission	4.78	0.76	5.00	0.00	4.80	0.83	0.351
Enforcing the lithotomy position	3.30	1.25	3.00	1.67	4.22	0.94	≤0.001
Allowing accompaniment during the second stage	2.99	1.55	2.71	1.63	2.76	1.66	0.691
Performing routine episiotomy	4.39	0.98	4.40	1.07	4.24	1.03	0.448
Saying “You do not know how to push”	4.72	0.80	5.00	0.00	4.76	0.84	0.238
Performing the Kristeller manoeuvre	4.60	0.75	3.92	1.16	4.00	1.09	≤0.001
Performing an episiotomy without anaesthesia	4.46	1.01	3.89	1.05	4.17	1.61	0.046
Prohibiting eating and drinking	3.54	1.12	3.31	1.54	3.93	1.02	0.108
Not providing covering/heating during delivery	4.40	0.87	4.62	0.65	4.59	0.88	0.252
Saying “Stop complaining; it is not that bad”	4.75	0.77	5.00	0.00	4.78	0.84	0.272
Not letting the woman shout	4.48	0.93	4.98	0.28	4.50	1.04	0.193
Performing a caesarean section due to slow dilation	4.00	1.14	4.23	0.92	4.33	0.96	0.249
Performing an emergency caesarean section without consent	4.33	1.11	4.69	0.63	4.54	0.95	0.376
Not allowing accompaniment in cases of instrumentation or caesarean section	4.54	0.79	4.08	1.11	4.17	0.90	0.012
Immediately cutting the cord	3.60	1.26	2.82	1.40	4.15	1.03	0.003
Suturing a tear without anaesthesia	4.37	0.94	4.58	0.66	4.48	0.96	0.588
Separating the mother and newborn	4.88	0.60	5.00	0.00	4.80	0.83	0.646
Allowing skin-to-skin contact after the paediatric examination	3.93	1.34	3.15	1.51	4.00	1.26	0.119
Taking the baby to the nursery	3.85	1.12	3.15	1.14	4.11	1.05	0.015
Giving formula without the mother’s consent	4.72	0.81	4.92	0.27	4.78	0.84	0.482

^1^ Kruskal–Wallis test.
